# 2-[(4-Chloro­phen­yl)(hy­droxy)meth­yl]-5,5-dimethyl-1,3,2-dioxaphosphinan-2-one

**DOI:** 10.1107/S1600536811008944

**Published:** 2011-03-19

**Authors:** Chubei Wang, Hao Peng, Hongwu He

**Affiliations:** a Key Laboratory of Pesticide and Chemical Biology, College of Chemistry, Central China Normal University, Wuhan 430079, People’s Republic of China.

## Abstract

In the title compound, C_12_H_16_ClO_4_P, the phospho­nate ring adopts a chair conformation. In the crystal, intermolecular O—H⋯O hydrogen bonds link the molecules into chains propagating along the *b* axis.

## Related literature

For the synthesis of hy­droxy­phospho­nates, see: Zhou *et al.* (2008 ▶). For the synthesis and biological activity of hy­droxy­phospho­nate derivatives, see: Peng *et al.* (2007 ▶); Liu *et al.* (2006 ▶). For standard bond lengths, see: Allen *et al.* (1987 ▶). 
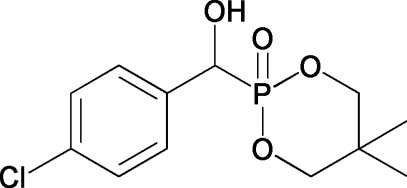


## Experimental

### 

#### Crystal data

C_12_H_16_ClO_4_P
*M* *_r_* = 290.67
Monoclinic, 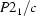

*a* = 12.8965 (11) Å
*b* = 9.4449 (8) Å
*c* = 11.6425 (10) Å
β = 98.630 (1)°
*V* = 1402.1 (2) Å^3^
*Z* = 4
Mo *K*α radiation
μ = 0.39 mm^−1^
*T* = 298 K
0.23 × 0.16 × 0.12 mm

#### Data collection

Bruker SMART APEX CCD area-detector diffractometer
10123 measured reflections
3463 independent reflections
3141 reflections with *I* > 2σ(*I*)
*R*_int_ = 0.071

#### Refinement

*R*[*F* ^2^ > 2σ(*F* ^2^)] = 0.062
*wR*(*F* ^2^) = 0.154
*S* = 1.15
3463 reflections
166 parameters
H-atom parameters constrained
Δρ_max_ = 0.72 e Å^−3^
Δρ_min_ = −0.28 e Å^−3^

### 

Data collection: *SMART* (Bruker, 2001 ▶); cell refinement: *SAINT-Plus* (Bruker, 2001 ▶); data reduction: *SAINT-Plus*; program(s) used to solve structure: *SHELXS97* (Sheldrick, 2008 ▶); program(s) used to refine structure: *SHELXL97* (Sheldrick, 2008 ▶); molecular graphics: *PLATON* (Spek, 2009 ▶); software used to prepare material for publication: *PLATON*.

## Supplementary Material

Crystal structure: contains datablocks global, I. DOI: 10.1107/S1600536811008944/wn2424sup1.cif

Structure factors: contains datablocks I. DOI: 10.1107/S1600536811008944/wn2424Isup2.hkl

Additional supplementary materials:  crystallographic information; 3D view; checkCIF report

## Figures and Tables

**Table 1:** Hydrogen-bond geometry (Å, °)

*D*—H⋯*A*	*D*—H	H⋯*A*	*D*⋯*A*	*D*—H⋯*A*
O4—H4⋯O3^i^	0.82	1.89	2.705 (3)	172

Symmetry code: (i) 
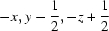
.

**Fig. 1.**
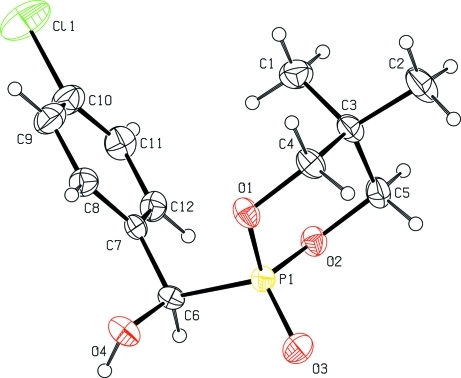
Molecular structure of the title compound, with displacement parameters drawn at the 50% probability level. Hydrogen atoms are shown as spheres of arbitrary radius.

**Fig. 2.**
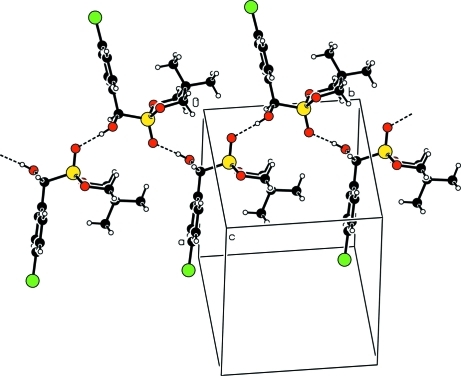
Part of the crystal packing, showing the intermolecular hydrogen bonds as dashed lines.

## supplementary crystallographic information

### Comment

Acyclic alpha-hydroxyphosphonates and cyclic alpha-hydroxyphosphonates can be used as very convenient intermediates. They are also an attractive class of biologically active compounds (Peng *et al.*, 2007; Liu *et al.*, 2006). In our research work aimed at searching for novel agrochemicals, we have attempted to synthesize hydroxyphosphonate derivatives using literature procedures. Here we report the crystal structure of the title compound (Fig. 1). The bond lengths (Allen *et al.*, 1987) and angles show normal values. The crystal structure is stabilized by intermolecular O—H···O hydrogen bonds (Table 1, Fig. 2).

### Experimental

The title compound was prepared according to literature procedures (Zhou *et al.* 2008). 4-Chlorobenzaldehyde (10 mmol) was added to a solution of 5,5-dimethyl-1,3,2-dioxaphosphinane (10 mmol) in triethylamine  (10 mmol). The mixture was stirred at room temperature for 20 h. Pure  title compound was afforded by column chromatography on silica gel (acetone/petroleum ether 1:2). Recrystallization from ethyl acetate over a period of one week gave colourless crystals of the title compound.

### Refinement

C-bound H atoms and the O-bound H atom were geometrically positioned (C—H 0.93–0.97 Å, O—H = 0.82 Å) and refined as riding, with *U*_iso_(H) =k*U*_eq_(C, O), where k = 1.5 for methyl H and OH and 1.2 for other H atoms.

### Crystal data

**Table:**

C_12_H_16_ClO_4_P	*F*(000) = 608
*M**_r_* = 290.67	*D*_x_ = 1.377 Mg m^−^^3^
Monoclinic, *P*2_1_/*c*	Mo *K*α radiation, λ = 0.71073 Å
*a* = 12.8965 (11) Å	Cell parameters from 4935 reflections
*b* = 9.4449 (8) Å	θ = 2.7–28.2°
*c* = 11.6425 (10) Å	µ = 0.39 mm^−^^1^
β = 98.630 (1)°	*T* = 298 K
*V* = 1402.1 (2) Å^3^	Block, colourless
*Z* = 4	0.23 × 0.16 × 0.12 mm

### Data collection

**Table:**

Bruker SMART APEX CCD area-detector diffractometer	3141 reflections with *I* > 2σ(*I*)
Radiation source: fine-focus sealed tube	*R*_int_ = 0.071
graphite	θ_max_ = 28.3°, θ_min_ = 2.7°
φ and ω scans	*h* = −13→17
10123 measured reflections	*k* = −9→12
3463 independent reflections	*l* = −15→15

### Refinement

**Table:**

Refinement on *F*^2^	Primary atom site location: structure-invariant direct methods
Least-squares matrix: full	Secondary atom site location: difference Fourier map
*R*[*F*^2^ > 2σ(*F*^2^)] = 0.062	Hydrogen site location: inferred from neighbouring sites
*wR*(*F*^2^) = 0.154	H-atom parameters constrained
*S* = 1.15	*w* = 1/[σ^2^(*F*_o_^2^) + (0.0581*P*)^2^ + 0.7742*P*] where *P* = (*F*_o_^2^ + 2*F*_c_^2^)/3
3463 reflections	(Δ/σ)_max_ < 0.001
166 parameters	Δρ_max_ = 0.72 e Å^−^^3^
0 restraints	Δρ_min_ = −0.28 e Å^−^^3^

### Special details

**Table:**

Geometry. All e.s.d.'s (except the e.s.d. in the dihedral angle between two l.s. planes) are estimated using the full covariance matrix. The cell e.s.d.'s are taken into account individually in the estimation of e.s.d.'s in distances, angles and torsion angles; correlations between e.s.d.'s in cell parameters are only used when they are defined by crystal symmetry. An approximate (isotropic) treatment of cell e.s.d.'s is used for estimating e.s.d.'s involving l.s. planes.
Refinement. Refinement of *F*^2^ against ALL reflections. The weighted *R*-factor *wR* and goodness of fit *S* are based on *F*^2^, conventional *R*-factors *R* are based on *F*, with *F* set to zero for negative *F*^2^. The threshold expression of *F*^2^ > σ(*F*^2^) is used only for calculating *R*-factors(gt) *etc*. and is not relevant to the choice of reflections for refinement. *R*-factors based on *F*^2^ are statistically about twice as large as those based on *F*, and *R*- factors based on ALL data will be even larger.

### Fractional atomic coordinates and isotropic or equivalent isotropic displacement parameters (Å^2^)

**Table:**

	*x*	*y*	*z*	*U*_iso_*/*U*_eq_	
C1	0.2944 (2)	0.3005 (3)	0.5354 (2)	0.0543 (6)	
H1A	0.2923	0.3256	0.6150	0.081*	
H1B	0.3596	0.3312	0.5135	0.081*	
H1C	0.2883	0.1996	0.5267	0.081*	
C2	0.2096 (3)	0.5330 (3)	0.4720 (3)	0.0635 (8)	
H2A	0.1547	0.5765	0.4188	0.095*	
H2B	0.2764	0.5659	0.4561	0.095*	
H2C	0.2014	0.5577	0.5501	0.095*	
C3	0.2035 (2)	0.3720 (2)	0.4576 (2)	0.0407 (5)	
C4	0.2059 (2)	0.3377 (2)	0.3303 (2)	0.0430 (5)	
H4A	0.1519	0.3917	0.2823	0.052*	
H4B	0.2733	0.3655	0.3099	0.052*	
C5	0.09896 (19)	0.3251 (2)	0.4897 (2)	0.0429 (5)	
H5A	0.0971	0.3465	0.5708	0.052*	
H5B	0.0428	0.3773	0.4434	0.052*	
C6	0.12155 (17)	−0.0698 (2)	0.36460 (19)	0.0359 (4)	
H6	0.0632	−0.1171	0.3939	0.043*	
C7	0.21964 (16)	−0.09498 (19)	0.44979 (18)	0.0330 (4)	
C8	0.31652 (19)	−0.1076 (3)	0.4118 (2)	0.0432 (5)	
H8	0.3212	−0.0978	0.3333	0.052*	
C9	0.4058 (2)	−0.1346 (3)	0.4902 (3)	0.0550 (6)	
H9	0.4705	−0.1435	0.4647	0.066*	
C10	0.3983 (2)	−0.1482 (3)	0.6055 (3)	0.0554 (7)	
C11	0.3035 (2)	−0.1347 (3)	0.6457 (2)	0.0509 (6)	
H11	0.2996	−0.1430	0.7245	0.061*	
C12	0.21483 (19)	−0.1088 (2)	0.5673 (2)	0.0422 (5)	
H12	0.1505	−0.1004	0.5936	0.051*	
Cl1	0.51070 (8)	−0.18286 (16)	0.70446 (9)	0.1062 (4)	
O1	0.18909 (13)	0.18663 (16)	0.30702 (13)	0.0403 (4)	
O2	0.08252 (12)	0.17262 (17)	0.46986 (13)	0.0402 (4)	
O3	−0.00798 (14)	0.1404 (2)	0.26116 (16)	0.0537 (5)	
O4	0.12788 (16)	−0.12340 (19)	0.25263 (15)	0.0526 (5)	
H4	0.0926	−0.1957	0.2418	0.079*	
P1	0.08858 (4)	0.11632 (6)	0.34372 (5)	0.03362 (17)	

### Atomic displacement parameters (Å^2^)

**Table:**

	*U*^11^	*U*^22^	*U*^33^	*U*^12^	*U*^13^	*U*^23^
C1	0.0489 (14)	0.0561 (16)	0.0541 (14)	−0.0019 (12)	−0.0045 (11)	0.0008 (12)
C2	0.088 (2)	0.0332 (12)	0.0674 (17)	−0.0063 (13)	0.0042 (16)	−0.0104 (12)
C3	0.0505 (13)	0.0286 (10)	0.0416 (11)	−0.0025 (9)	0.0024 (10)	−0.0031 (8)
C4	0.0560 (14)	0.0298 (10)	0.0439 (12)	−0.0069 (10)	0.0099 (10)	0.0024 (9)
C5	0.0511 (13)	0.0373 (11)	0.0411 (11)	0.0065 (10)	0.0093 (10)	−0.0078 (9)
C6	0.0345 (10)	0.0291 (9)	0.0438 (11)	−0.0071 (8)	0.0048 (8)	−0.0022 (8)
C7	0.0356 (10)	0.0188 (8)	0.0442 (11)	−0.0014 (7)	0.0045 (8)	0.0004 (7)
C8	0.0402 (12)	0.0439 (12)	0.0463 (12)	−0.0019 (10)	0.0096 (10)	0.0011 (10)
C9	0.0366 (12)	0.0619 (17)	0.0668 (16)	0.0031 (11)	0.0087 (11)	0.0032 (13)
C10	0.0442 (14)	0.0571 (16)	0.0606 (16)	0.0052 (12)	−0.0060 (11)	0.0079 (13)
C11	0.0615 (16)	0.0483 (14)	0.0421 (12)	0.0045 (12)	0.0049 (11)	0.0084 (10)
C12	0.0419 (12)	0.0384 (12)	0.0476 (12)	0.0039 (9)	0.0114 (10)	0.0043 (9)
Cl1	0.0629 (6)	0.1541 (11)	0.0912 (7)	0.0175 (6)	−0.0221 (5)	0.0266 (7)
O1	0.0520 (9)	0.0310 (8)	0.0412 (8)	−0.0050 (7)	0.0175 (7)	−0.0022 (6)
O2	0.0435 (9)	0.0373 (8)	0.0423 (8)	0.0009 (7)	0.0150 (7)	−0.0016 (6)
O3	0.0485 (10)	0.0528 (10)	0.0546 (10)	0.0105 (8)	−0.0089 (8)	0.0022 (8)
O4	0.0622 (12)	0.0444 (10)	0.0503 (10)	−0.0086 (8)	0.0054 (8)	−0.0130 (8)
P1	0.0342 (3)	0.0304 (3)	0.0358 (3)	0.0021 (2)	0.0039 (2)	0.0004 (2)

### Geometric parameters (Å, °)

**Table:**

C1—C3	1.528 (3)	C6—P1	1.816 (2)
C1—H1A	0.9600	C6—H6	0.9800
C1—H1B	0.9600	C7—C12	1.385 (3)
C1—H1C	0.9600	C7—C8	1.392 (3)
C2—C3	1.530 (3)	C8—C9	1.382 (3)
C2—H2A	0.9600	C8—H8	0.9300
C2—H2B	0.9600	C9—C10	1.367 (4)
C2—H2C	0.9600	C9—H9	0.9300
C3—C5	1.518 (3)	C10—C11	1.379 (4)
C3—C4	1.522 (3)	C10—Cl1	1.742 (3)
C4—O1	1.463 (3)	C11—C12	1.373 (3)
C4—H4A	0.9700	C11—H11	0.9300
C4—H4B	0.9700	C12—H12	0.9300
C5—O2	1.469 (3)	O1—P1	1.5716 (16)
C5—H5A	0.9700	O2—P1	1.5748 (16)
C5—H5B	0.9700	O3—P1	1.4721 (18)
C6—O4	1.412 (3)	O4—H4	0.8200
C6—C7	1.505 (3)		
			
C3—C1—H1A	109.5	C7—C6—P1	113.47 (14)
C3—C1—H1B	109.5	O4—C6—H6	108.2
H1A—C1—H1B	109.5	C7—C6—H6	108.2
C3—C1—H1C	109.5	P1—C6—H6	108.2
H1A—C1—H1C	109.5	C12—C7—C8	118.8 (2)
H1B—C1—H1C	109.5	C12—C7—C6	120.54 (19)
C3—C2—H2A	109.5	C8—C7—C6	120.7 (2)
C3—C2—H2B	109.5	C9—C8—C7	120.4 (2)
H2A—C2—H2B	109.5	C9—C8—H8	119.8
C3—C2—H2C	109.5	C7—C8—H8	119.8
H2A—C2—H2C	109.5	C10—C9—C8	119.4 (2)
H2B—C2—H2C	109.5	C10—C9—H9	120.3
C5—C3—C4	109.08 (19)	C8—C9—H9	120.3
C5—C3—C1	110.8 (2)	C9—C10—C11	121.5 (2)
C4—C3—C1	110.9 (2)	C9—C10—Cl1	119.5 (2)
C5—C3—C2	107.2 (2)	C11—C10—Cl1	119.0 (2)
C4—C3—C2	108.0 (2)	C12—C11—C10	118.9 (2)
C1—C3—C2	110.6 (2)	C12—C11—H11	120.6
O1—C4—C3	111.34 (17)	C10—C11—H11	120.6
O1—C4—H4A	109.4	C11—C12—C7	121.1 (2)
C3—C4—H4A	109.4	C11—C12—H12	119.4
O1—C4—H4B	109.4	C7—C12—H12	119.4
C3—C4—H4B	109.4	C4—O1—P1	117.87 (14)
H4A—C4—H4B	108.0	C5—O2—P1	116.83 (14)
O2—C5—C3	111.10 (17)	C6—O4—H4	109.5
O2—C5—H5A	109.4	O3—P1—O1	114.12 (11)
C3—C5—H5A	109.4	O3—P1—O2	113.65 (10)
O2—C5—H5B	109.4	O1—P1—O2	105.62 (9)
C3—C5—H5B	109.4	O3—P1—C6	113.27 (10)
H5A—C5—H5B	108.0	O1—P1—C6	105.02 (9)
O4—C6—C7	113.10 (18)	O2—P1—C6	104.20 (9)
O4—C6—P1	105.57 (15)		
			
C5—C3—C4—O1	−57.7 (3)	C10—C11—C12—C7	−0.6 (4)
C1—C3—C4—O1	64.7 (3)	C8—C7—C12—C11	−0.1 (3)
C2—C3—C4—O1	−173.9 (2)	C6—C7—C12—C11	178.7 (2)
C4—C3—C5—O2	58.9 (2)	C3—C4—O1—P1	54.0 (2)
C1—C3—C5—O2	−63.5 (2)	C3—C5—O2—P1	−56.2 (2)
C2—C3—C5—O2	175.7 (2)	C4—O1—P1—O3	80.51 (18)
O4—C6—C7—C12	−151.46 (19)	C4—O1—P1—O2	−45.05 (18)
P1—C6—C7—C12	88.3 (2)	C4—O1—P1—C6	−154.86 (16)
O4—C6—C7—C8	27.3 (3)	C5—O2—P1—O3	−79.99 (18)
P1—C6—C7—C8	−92.9 (2)	C5—O2—P1—O1	45.86 (17)
C12—C7—C8—C9	0.5 (3)	C5—O2—P1—C6	156.25 (16)
C6—C7—C8—C9	−178.2 (2)	O4—C6—P1—O3	56.16 (18)
C7—C8—C9—C10	−0.3 (4)	C7—C6—P1—O3	−179.45 (15)
C8—C9—C10—C11	−0.4 (5)	O4—C6—P1—O1	−69.01 (15)
C8—C9—C10—Cl1	179.8 (2)	C7—C6—P1—O1	55.38 (17)
C9—C10—C11—C12	0.8 (4)	O4—C6—P1—O2	−179.83 (14)
Cl1—C10—C11—C12	−179.3 (2)	C7—C6—P1—O2	−55.44 (17)

### Hydrogen-bond geometry (Å, °)

**Table:**

*D*—H···*A*	*D*—H	H···*A*	*D*···*A*	*D*—H···*A*
O4—H4···O3^i^	0.82	1.89	2.705 (3)	172

Symmetry codes: (i) −*x*, *y*−1/2, −*z*+1/2.
